# Non-Coding RNAs in Health and Disease: Editorial

**DOI:** 10.3390/biomedicines11010014

**Published:** 2022-12-21

**Authors:** Giuseppina Catanzaro

**Affiliations:** Department of Experimental Medicine, Sapienza University of Rome, 00161 Rome, Italy; giuseppina.catanzaro@uniroma1.it

Non-coding RNAs (ncRNAs) represent the largest part of the transcriptional production of the human genome and play key roles in health and disease processes. They are involved in regulatory functions and interact with each other to regulate several cellular pathways. Thus, the perturbation of their interactions, as well as their dysregulation, may have striking consequences. In addition, ncRNAs have also been described in the extracellular compartment as being able to mediate cell-to-cell communication and to act as disease-specific diagnostic, prognostic, and predictive biomarkers.

The papers published in this Special Issue address the roles of ncRNAs in different backgrounds.

Literature reviews deepened the knowledge of the significance of sex differences in ncRNAs’ expression and function, as a way to understand their role in pregnancy and related disorders [1] as well as their role as promising biomarkers and/or therapeutic targets in the evaluation of infertility and embryo quality [2]. Looking at diseases, Filardi et al. focused on the existence of dietary supplements, such as polyphenols and other plant-derived molecules, with effects on the expression of microRNAs dysregulated by obesity and the consequent favorable activity on the pathophysiological mechanisms underlying obesity [3]. Maier and Maier focused instead on the possible role of both microRNAs and long ncRNAs (lncRNAs) as potential circulating diagnostic biomarkers for endometriosis and as a way to differentiate endometriosis stages [4], whereas Stasevich et al. conducted an in-depth literature analysis of the ncRNAs that can potentially be used in specific tumors, since they act as MYC proto-oncogenes regulators [5].

Additionally, original articles concentrated on different aspects of the biology of ncRNAs. Gargaun et al. looked at the possible role of the lncRNA 44s2 and of 45-55 multi-exon skipping as therapeutic strategies in Duchenne and Becker muscular dystrophies [6], while Treeck et al. identified DSCAM-AS1, a lncRNA with oncogenic roles in endometrial adenocarcinoma, as witnessed by its association with a shorter overall survival of patients affected by this cancer and with the expression of both the endometrial tumor promotor gene *prolactin* (*PRL)* and the Estrogen Receptor α (ERα) [7].

Other authors have focused on microRNAs. Specifically, Scalavino et al. described a role of miR-195-5p in the regulation of intestinal tight junctions’ through the modulation of its target, claudin-2, which is involved in barrier function. Thus, miR-195-5p represents a potential therapeutic target in inflammatory bowel disease [8]. Matarese et al. instead focused on COVID-19 and identified a microRNA, miR-98-5p, that specifically targets the transmembrane protease serine 2 (TMPRSS2), one of the two co-factors used by the coronavirus to access human host cells. Specifically, the authors suggested that the targeting of miR-98-5p may be used as a therapeutic strategy against COVID-19 [9]. Other authors concentrated on circulating microRNAs as biomarkers of disease. Specifically, Yavropoulou et al. compared the expression of seven microRNAs circulating in the serum of patients affected by chronic kidney disease-mineral and bone disorder (CKD-MBD) and healthy gender and age-matched subjects. They demonstrated that the serum levels of miR-21-5p, miR-124-3p, and miR-23a-3p, the first two microRNAs related to osteoblasts and the latter to osteoclasts, were significantly dysregulated in CKD-MBD patients. In addition, miR-124-3p showed high sensitivity and specificity in the identification of CKD-MBD patients affected by osteoporosis [10]. Finally, Panebianco et al. integrated tissue and plasma-circulating microRNA profiles with MRI biomarkers and clinical data to improve the early detection of clinically significant prostate cancer (csPCa) and to propose the combination of molecular data and MRI parameters as a prebiopsy triage for patients at risk of csPCa. Three plasma circulating microRNAs, namely miR-548a-3p, miR-138-5p, and miR-520d-3p, were identified as candidate biomarkers, and their up-regulation in plasma of csPCa patients was validated in a larger cohort of samples. The addition of the three microRNAs to MRI biomarkers could ameliorate patients’ detection and risk stratification, reducing overdiagnosis and consequently the number of unnecessary biopsies and their related costs [11].

Given all these contributions, it is not only clear that the ncRNAs play a pivotal role in human health, but also that the deepening of their biological role and of their possible use as biomarkers will open new interesting perspectives for clinicians to reach early diagnosis and prognosis and to propose tailored therapeutic strategies, designed to maximize the efficacy and minimize unwanted side effects, making them the best candidates to render precision medicine a reality.

## Data Availability

Data sharing not applicable.
